# Amazon savannization and climate change are projected to increase dry season length and temperature extremes over Brazil

**DOI:** 10.1038/s41598-024-55176-5

**Published:** 2024-03-01

**Authors:** Marcus Jorge Bottino, Paulo Nobre, Emanuel Giarolla, Manoel Baptista da Silva Junior, Vinicius Buscioli Capistrano, Marta Malagutti, Jonas Noboru Tamaoki, Beatriz Fátima Alves de Oliveira, Carlos Afonso Nobre

**Affiliations:** 1 National Institute for Space Research - INPE, Rodovia Presidente Dutra SP-RJ Km 40, Cachoeira Paulista, São Paulo 12630-000 Brazil; 2 Federal University of Mato Grosso Do Sul (UFMS), Campo Grande, Mato Grosso do Sul Brasil; 3 Fiocruz Regional Office of Piauí, National School of Public Health, Oswaldo Cruz Foundation, Teresina, Piauí Brazil; 4 Institute of Advanced Studies (IEA), São Paulo University, São Paulo, São Paulo Brazil

**Keywords:** Climate sciences, Climate change, Climate and Earth system modelling

## Abstract

Land use change and atmospheric composition, two drivers of climate change, can interact to affect both local and remote climate regimes. Previous works have considered the effects of greenhouse gas buildup in the atmosphere and the effects of Amazon deforestation in atmospheric general circulation models. In this study, we investigate the impacts of the Brazilian Amazon savannization and global warming in a fully coupled ocean-land-sea ice-atmosphere model simulation. We find that both savannization and global warming individually lengthen the dry season and reduce annual rainfall over large tracts of South America. The combined effects of land use change and global warming resulted in a mean annual rainfall reduction of 44% and a dry season length increase of 69%, when averaged over the Amazon basin, relative to the control run. Modulation of inland moisture transport due to savannization shows the largest signal to explain the rainfall reduction and increase in dry season length over the Amazon and Central-West. The combined effects of savannization and global warming resulted in maximum daily temperature anomalies, reaching values of up to 14 °C above the current climatic conditions over the Amazon. Also, as a consequence of both climate drivers, both soil moisture and surface runoff decrease over most of the country, suggesting cascading negative future impacts on both agriculture production and hydroelectricity generation.

## Introduction

In addition to being the habitat of a great number of vegetal and animal species^1^, the Amazon rainforest is also known to be an important player in the global climatic system. Several works have demonstrated its role in modulating rainfall and air temperature, both locally (e.g.,^2–6^) and remotely^2,7,8^. One role of the Amazon rainforest is to regulate the hydrological cycle both over the forest itself and in distant areas^9,10^. Via intense evapotranspiration, the tropical forest pumps latent heat deep into the atmosphere to balance the strong surface radiative heating^11^. Furthermore, moisture recycling processes are an important mechanism for the advection of water vapor towards the interior of the continent^12,13,14^. Water vapor originating from the tropical Atlantic Ocean is transported over South America by the Trade Winds, feeding into the precipitation processes over the Amazon basin^12,15,16^. A portion of the transported water vapor reaches the western portion of the basin with replenishing water vapor content supplied by Amazon rainforest evapotranspiration^11^. However, we have yet to investigate to what extent two competing factors induce rainfall reductions over the Amazon Basin and elsewhere in South America, namely savannization (i.e. the substitution of the original Amazonian broadleaf evergreen trees by broadleaf trees with ground cover) and global warming. Additionally, it is of interest to gauge the dependence of the agricultural growing season in Central-West and elsewhere in Southern Brazil on the upstream water vapor transport contribution from the Amazon rainforest^17^. A study using satellite-based and rain gauge observations from 1981 to 2019 found that a large fraction of this agriculturally important region has experienced reduced dry season rainfall^18^. Over the state of Rondônia in Brazil, water vapor originating from ocean evaporation accounts for 58% of the mean dry season precipitation while continental recycling contributes 42%^19^. One mechanism of moisture transport from the Amazon southward is the South American Low-Level Jet (SALLJ), a narrow northerly wind speed maximum present just above the atmospheric surface boundary layer^9^. The SALLJ is an important component of tropical-extratropical heat and moisture exchange in South America and can favor deep moist convection in Southeastern South America^20^. According to Baker and Spracklen^21^, the CMIP6 models underestimate the effects of vegetation on precipitation.

The resilience of the Amazon rainforest to climate change and savannization is crucial for biodiversity, regional climate stability, and the global carbon cycle^6^. Savannization and climate change, via increasing dry-season length and drought frequency, might have already pushed the Amazon close to a critical threshold of rainforest dieback^22^. Increases in the length of the dry season have been reported in several recent studies^23–26^.

As a consequence of being a rainforest, the Amazon rainforest is highly averse to fire; therefore, fires occur in the Amazon only under specific climatic conditions (i.e., warm and dry) and after savannization^27,28^. The advance of human activities over the rainforest, which is a source of ignition, and global climate changes related to the increased concentration of greenhouse gasses (GHG) in the atmosphere may promote favorable conditions for increases in fire occurrence and severity in the Amazon^29^.

Since the industrial revolution, anthropic activities have increased the concentration of GHG in the atmosphere^30^. Such increases are aggravating the planetary greenhouse effect, which by itself is a naturally occurring process essential for the maintenance of life on Earth. The global average tropospheric temperature increases are proportional to atmospheric GHG concentration increases^31,32^. In the context of a pessimistic emission scenario RCP8.5 (Representative Concentration Pathways 8.5) for the end of the twenty-first century, climate models project an increase in the global mean air temperature of up to 5.7 °C^32^. The impacts of climate change are diverse, affecting both human activities^33^ and meteorological events; however, future projections of climate change show that under dire scenarios of GHG concentration increases, the Amazon rainforest could lose its natural barriers against fire, as the nights would become warmer and extended consecutive dry day periods would become more frequent^32,34^. The expected result of this interplay of processes is a contraction of the humid and dense Amazon rainforest, giving way to a Cerrado-like biome^35^. Acting synergistically with ongoing anthropic Amazon deforestation, the deterioration of forest integrity might increase climate change pressure in the region, especially endangering productive areas responsible for supporting global food security^36,37^. In addition, some authors suggest that the Amazon might have tipping points linked to exceeding savannization and temperature thresholds^38–41^.

The strong rainfall seasonality over the Amazon basin and the relatively rapid transition between the wet and dry seasons associated with the onset of the rainy season is related to the establishment of the South America Monsoon System (SAMS)^42^. The SAMS is controlled by large-scale thermodynamic conditions influenced by the near-equatorial sea surface temperature (SST)^43^. It has been suggested that land-surface dryness during the dry season is the main cause of the delayed onset of the subsequent wet season^27^. Projected future savannization scenarios over the Amazon show increases in the frequency of longer dry seasons^44,45^.

In this study, we investigate the impacts of both forest cover change scenarios over the Brazilian portion of the Amazon basin and global warming on the continental hydrology and temperature regimes. We specifically address the effects of dry season length, total rainfall, and temperature changes, as well as their combined effects, on soil moisture and surface runoff distributions over South America.

## Results and discussion

Three sets of numerical experiments utilizing the Brazilian Earth System Model-BESM version 2.5^46–49^ were performed to estimate the impacts of (1) Amazon rainforest cover change, (2) global warming, and (3) the combined effects of the previous experiments on the hydrological cycle and temperature over South America. The model experiments were labeled as follows: for GHG scenarios (Historical-Hi, and RCP8.5-R8) and for land use scenarios (Fo-Forested, and Sa-Savannah) (see Supplementary Fig. S1), generating a total of four combinatorial simulation runs. For example, HiSa represents the historical GHG scenario with the savannah land use pattern over the Amazon Basin, whereas R8Fo indicates the RCP8.5 scenario run with the Amazon rainforest land use pattern. The results presented are based on 28 simulation years, after a two-year spinup run. Total precipitation, daily maximum and minimum air temperatures at 2 m, surface runoff, soil moisture, and atmospheric profiles of wind and specific humidity at daily time scale are used for the following analyses.

### Annual precipitation change

**Figure 1:**
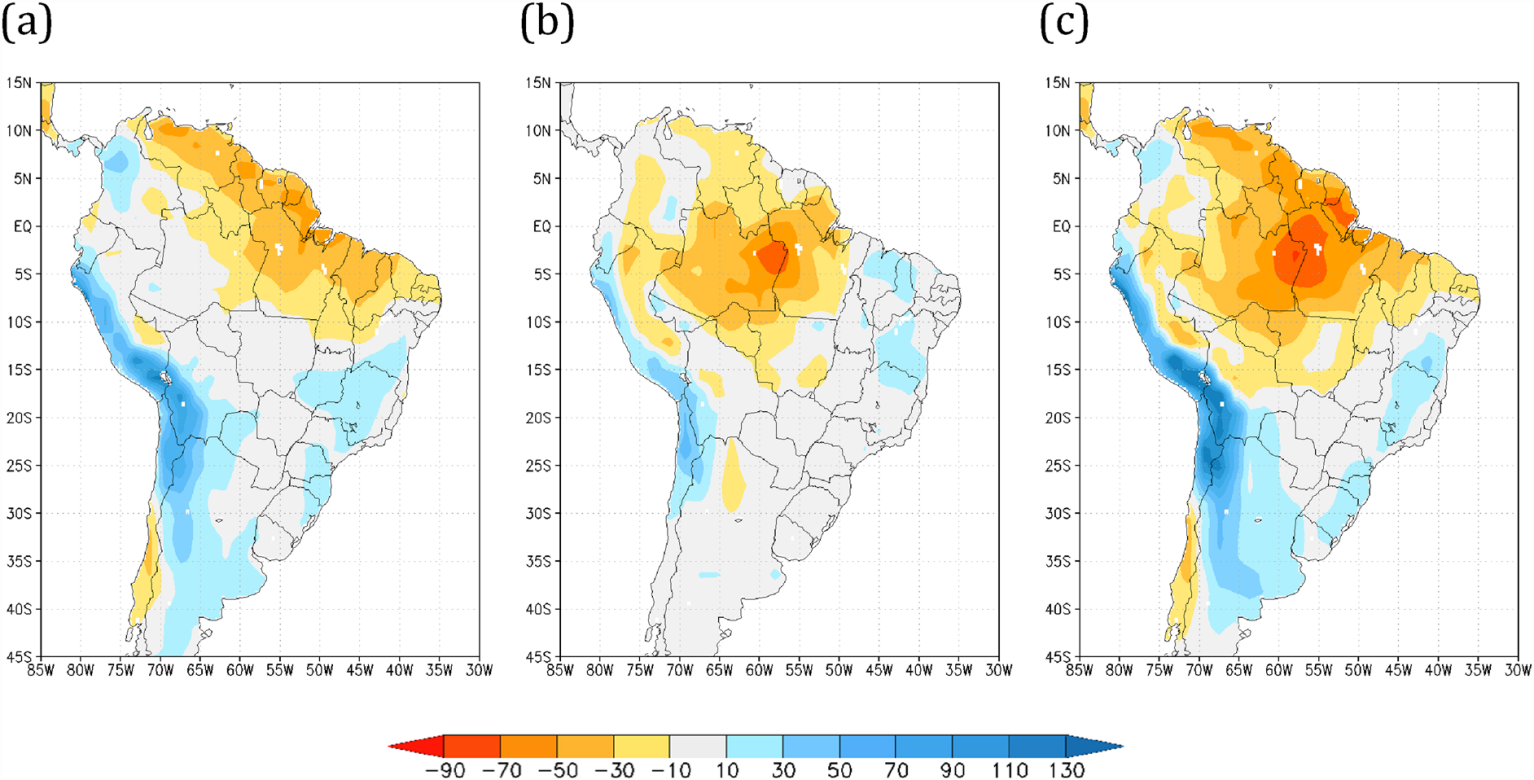
Annual mean precipitation differences relative to the HiFo control run (in %). Annual mean precipitation differences (in %) relative to the historical forested (HiFo, 1983–2010) control run for scenarios (**a**) RCP8.5 forested (R8Fo, 2073–2100), (**b**) historical savannah (HiSa, 1983–2010), and (**c**) RCP8.5 savannah (R8Sa, 2073–2100). Maps made by COLA GrADS v2.0.

The annual precipitation differences of the Amazon savannization and global warming scenarios relative to the HiFo control run are shown in Fig. 1. While the effects of the global warming scenario for the forested simulation (R8Fo) depict negative deviations in annual precipitation mostly over the northern-northeastern border of South America (Fig. 1a), the pattern of rainfall reduction expands inland when savannization is considered, both for current climate conditions HiSa (Fig. 1b) and the R8Sa future global warming climate scenario (Fig. 1c). The rainfall control run (HiFo) is shown in Supplementary Fig. S2. The severe rainfall reduction (up to 70%) over areas in central Amazon for the HiSa (Fig. 1b) is noteworthy; however, when both global warming and vegetation cover change are simultaneously applied, the region of the Amazon basin interior with a 70% total annual rainfall reduction extends further south over the Central-West Region (Fig. 1c). The precipitation reduction over the Amazon basin for the HiSa scenario agrees with previous Amazon deforestation studies^50,51^.

### Dry season length change

**Figure 2:**
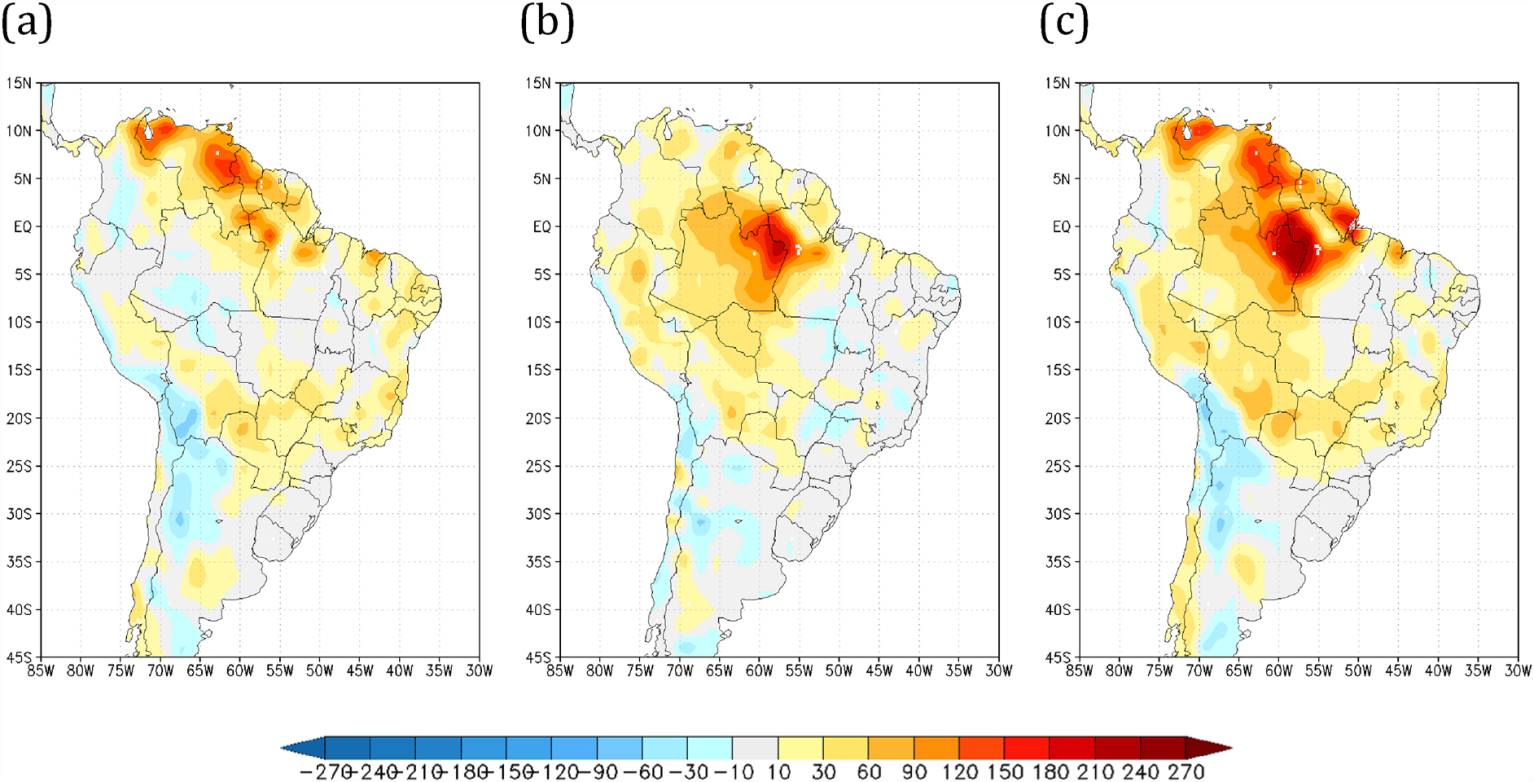
Dry season length differences relative to the HiFo control run (in days). The dry season defined by the length of the tail distribution of the largest 10% of the number of consecutive dry days relative to the historical forested (HiFo, 1983–2010) control run (in days) for scenarios (**a**) R8Fo, (**b**) HiSa, and (**c**) R8Sa. In yellow–red and bluish regions, the values are larger or shorter (respectively) compared with those in the HiFo simulation. Maps made by COLA GrADS v2.0.

The impact of land use and climate changes on the dry season length over Brazil was evaluated by examining the distribution of consecutive dry day events (dry season length) within the 28 years of numerical simulations (described in the Methods). To study the effects of both global warming and Amazon land cover change on dry season length, relative to HiFo scenario, a severe dry season length index (SDI) was defined as the length of consecutive dry days representing the longest 10% of the frequency distribution occurring over each grid point. Figure 2 shows the spatial distribution of the SDI for the deviations from the R8Fo, HiSa, and R8Sa scenarios relative to the HiFo simulation. It is noteworthy that while the larger effect of global warming on increasing the SDI are confined to the north-northeast portions of South America (Fig. 2a), the impacts of savannization over the Amazon extend the SDI southward over the interior portion of the Amazon and onward to southern Brazil (Fig. 2b). Furthermore, this effect is exacerbated when global warming is combined with Amazon savannization, increasing it by 60 days in the Amazon basin relative to the HiFo, and extending the length of the SDI to the central-southeastern regions of Brazil (Fig. 2c). The SDI spatial distribution for the control run (HiFo) is shown in Supplementary Fig. S3. According to previous studies^10,13,17^, changes in precipitation patterns over deforested areas in Amazonia is linked to a lack of moisture recycling and changes in atmospheric circulation reducing moisture convergence mechanisms^50^, which affect cascade moisture recycling along the way. Another study found that savannization over the Amazon basin increases the frequency of longer dry seasons in the central-southern Amazon (by 29% or 57%), depending on the savannization scenario considered^44^.

### Hydrological cycle change

**Figure 3:**
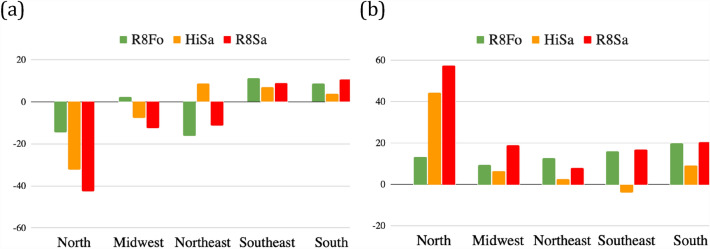
Regional average of annual mean precipitation and dry season length. (**a**) Annual mean precipitation differences relative to the HiFo control run (in %), and (**b**) dry season length (in %) change (expressed as the largest 10% number of consecutive dry days) averaged over each Region of Brazil for R8Fo (green), HiSa (orange), and R8Sa (red).

The bar chart in Fig. 3a shows the area-averaged annual rainfall deviations for each Region of Brazil relative to the HiFo control run. It is noteworthy that the negative annual rainfall anomalies over the Amazon and Central-West are the largest when both savannization and global climate change are considered, with the R8Sa presenting the largest rainfall reduction over both regions (see the orange and red bars in Fig. 3a). These results also suggest that in the Northeast region, global climate warming decreases rainfall, while under current climate conditions, savannization leads to a marginal rainfall increase. It is also notable that the South and Southeast regions experienced increased rainfall in all scenarios compared with the current climate, which is accompanied by the increased SDI (Fig. 3b).

The percent variation in the SDI relative to the HiFo experiment for the land use and global warming scenarios, averaged over each of the five regions of the country, is shown in Fig. 3b. The increased frequency of longer periods of consecutive dry days is notable in all regions for all R8 scenarios, with the largest dry season length increase occurring in the R8Sa experiment (red bars in Fig. 3b) for all regions. The drying effect of savannization, confirmed by comparing the R8Fo and R8Sa indices (the green and red bars in Fig. 3b, respectively), is most notable over the North, but can also be seen over the Central-est region.

### Meridional moisture advection

**Figure 4:**
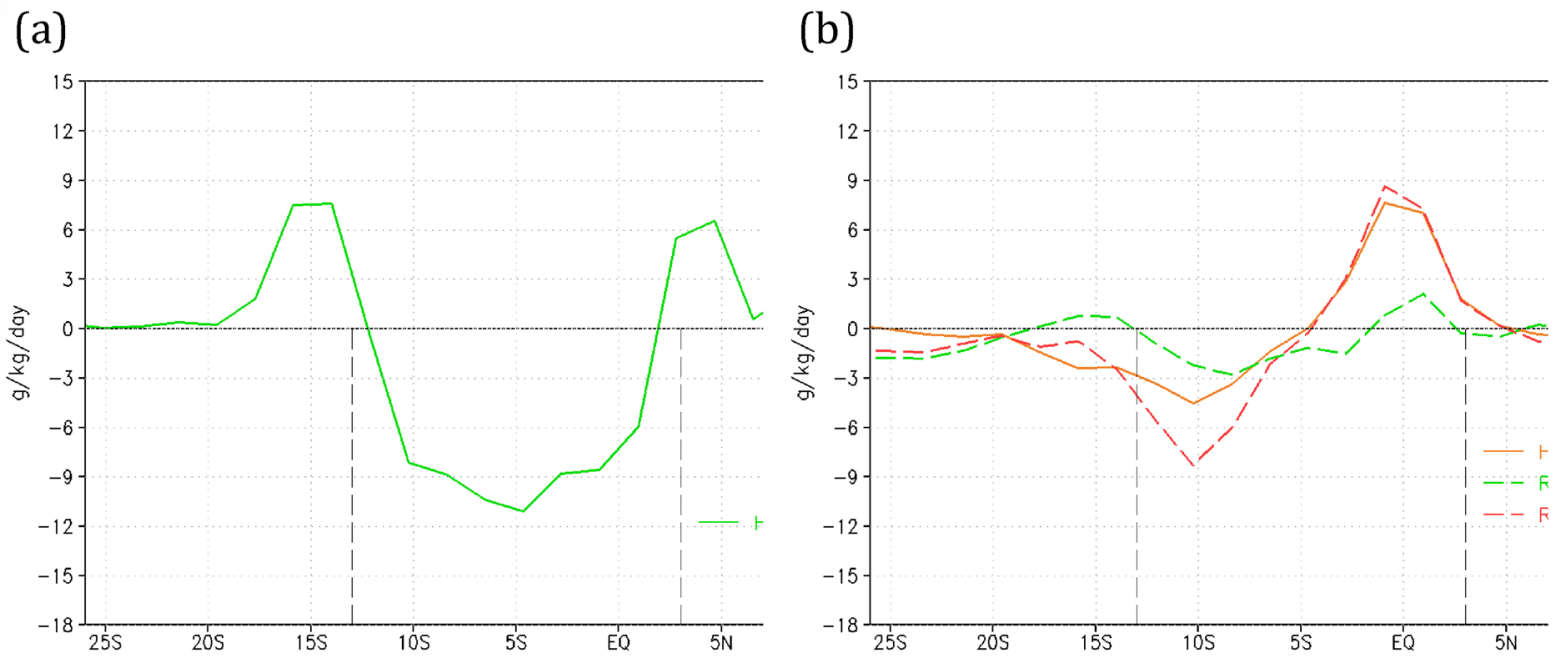
Low troposphere meridional humidity advection. DJFM mean, vertically integrated low troposphere (975–700 hPa) meridional humidity advection (in g/kg/day), for (**a**) HiFo and (**b**) deviations of the HiSa (continuous orange line), R8Fo (dashed green line), and R8Sa (dashed red line) relative to HiFo. The Amazon Basin latitude range is delimited by vertical black dashed lines. Negative advection indicates decreased humidity in the direction the wind is blowing.

As shown in the previous section, the replacement of the Amazon rainforest by savannah vegetation has produced a pronounced increase in the SDI, primarily in the Amazon region, but also extending to the central-southeastern parts of the continent. The SDI duration is invariantly enhanced by the effects of global warming. Other studies^10,15,52^ have presented evidence that reduced moisture in the basin affects distant regions of the continent via reduced southward moisture transport. The section of this flow lying adjacent to the Andes will, on some occasions, develop a core of particularly high-speed winds called the SALLJ^15,53^. This process is evaluated here by vertically integrating and averaging the low troposphere meridional moisture advection (between 975 and 700 hPa) over the area between longitudes 55°W and 65°W (see dashed rectangle in the Supplementary Fig. S1) during the summer season (DJFM) (Fig. 4). For the HiFo experiment, the region north of 3°N gains moisture from the Atlantic Ocean (indicated by the positive advection in Fig. 4a). Between 3°N and 12°S, delimiting the Amazon Basin (indicated by the vertical dashed lines in Fig. 4a), negative advection indicates that the region loses moisture to its neighboring southern region. This moisture is carried southward, beyond the Amazon Basin, to the latitude belt between 12°S and 20°S (positive advection). The R8Fo experiment does not show a significant impact on meridional moisture advection relative to the HiFo control case (see dashed green line in Fig. 4b), despite the increase in the specific humidity in the atmosphere due to the increase in global temperature (Supplementary Fig. S4b). However, when savannah vegetation is considered, a pronounced change in moisture advection relative to the HiFo control is observed. There is a significant reduction in negative advection over the northern portion of the Amazon Basin (positive difference, continuous red line in Fig. 4b), a result of less humidity available to advection (supplementary Fig. S4b). By contrast, there is negative advection intensification over the southern portion of the basin due to strengthened southward wind (supplementary Fig. S4d). This wind intensification is attributed to the reduced vegetation roughness associated with savannah cover. The reduction of positive advection south of the Amazon Basin indicates that the supply of moisture by the forest has decreased in this region. This reduced moisture advection is even more intense when driven by the joint effects of global warming and savannization (dashed red line in Fig. 4b), suggesting that the combined effects of savannization and global warming may induce a deficit of water vapor from the Amazon region in the regions south of the Amazon. The lack of rainfall in the Brazilian Pantanal during the summers of 2019 and 2020 was accompanied by reduced transport of warm and humid summer air from Amazonia^54^.

### Air temperature change

**Figure 5:**
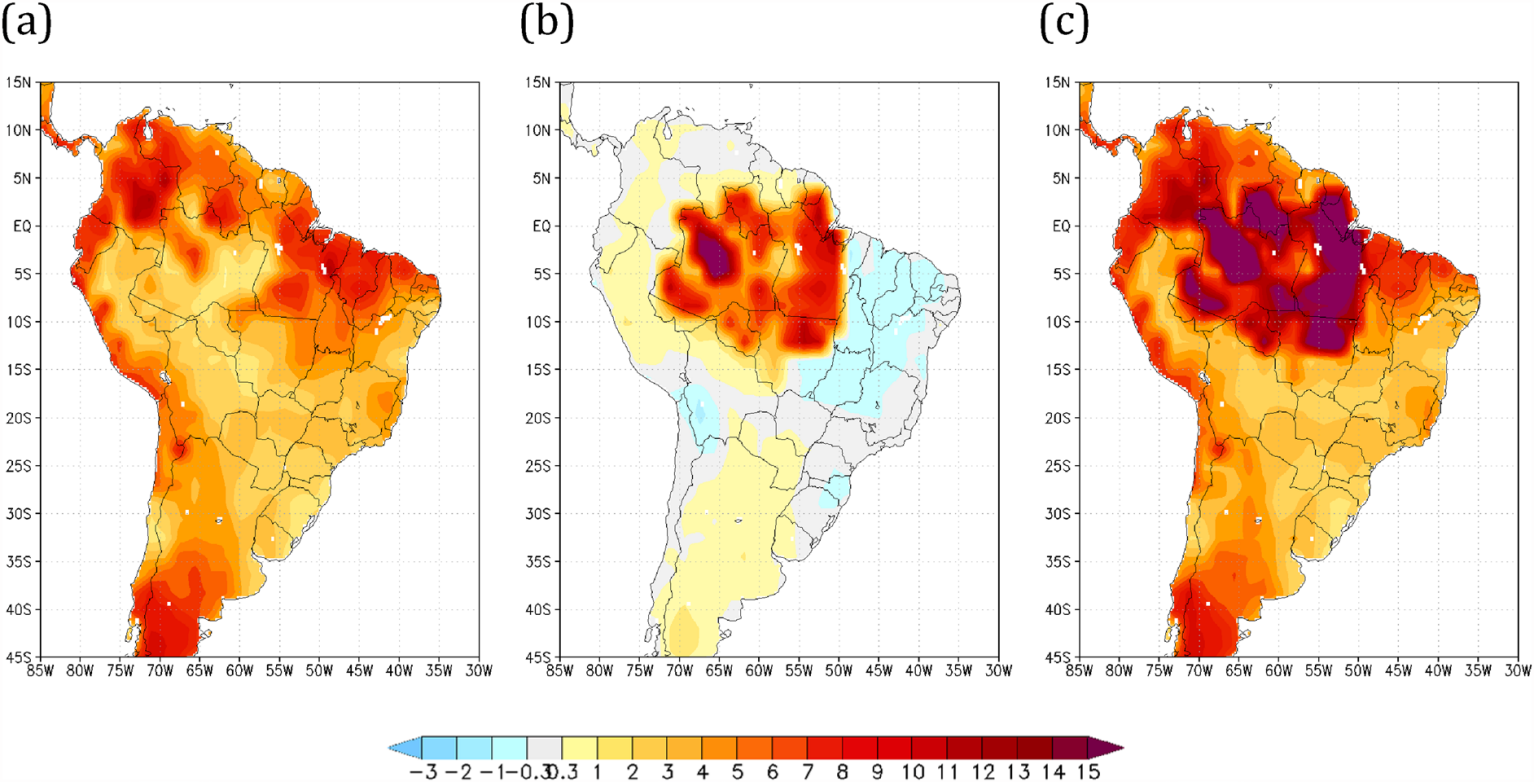
Climatological daily maximum air temperatures (in °C). Climatological daily maximum air temperatures (in °C) for the warmest month for the (**a**) R8Fo, (**b**) HiSa, and (**c**) R8Sa experiments relative to the HiFo simulation. Maps made by COLA GrADS v2.0.

To examine the impacts of global warming and savannization over the Amazon on the occurrence of air temperature extremes, the daily maximum temperature predicted at every grid point for each scenario was considered for the 28-year-long simulations. Figure 5 shows the maximum air temperature anomalies for the warmest months over South America for the R8Fo, HiSa, and R8Sa experiments relative to the HiFo control run. Consistent with other results^55,56^, the R8Fo experiment shows only positive anomalies over the continent (Fig. 5a), with the warmest deviations occurring over north-northeast and southern South America. By contrast, the effects of the savannization scenario for the current climate experiment HiSa (Fig. 5b) shows positive air temperature anomalies over the Amazon and negative anomalies eastward over the Northeast region. The combined effects of global warming and the savannization scenario in experiment R8Sa (Fig. 5c) shows the largest temperature anomalies, reaching values as high as 14 °C above the HiFo control run in central Amazon, which represents actual maximum temperatures surpassing 46 °C. The maximum air temperature for the HiFo control run is shown in supplementary Fig. S5. Experiment R8Fo produces an increment of 3.3 °C (average for the period 2073–2100) over the Amazon Basin (see Supplementary Fig. 1c), while experiment R8Sa presents an area average temperature increase of 5.4 °C over the Amazon relative to the HiFo control. These values lie within the same range obtained in a numerical experiment that investigated the combined effects of global warming and Amazon savannization using only atmospheric GCM^57^.

The seasonal thermal amplitude (STA) at each gridpoint, as measured by the difference between the daily maximum temperature of the warmest month and the daily minimum temperature of the coldest month, is shown in Supplementary Fig. S6. The average amplitude over the Amazon Basin is 13.5 °C for the HiFo. However, the STA increases 1.1 °C for the R8Fo, 9.0 °C for the HiSa, and 11.0 °C for the R8Sa relative to the HiFo experiment. The increase of the STA and the dry season length (Fig. 2) over the Amazon Basin might lead to adverse consequences for agriculture^39,58^ and human health^33^.

### Soil moisture and runoff changes

**Figure 6:**
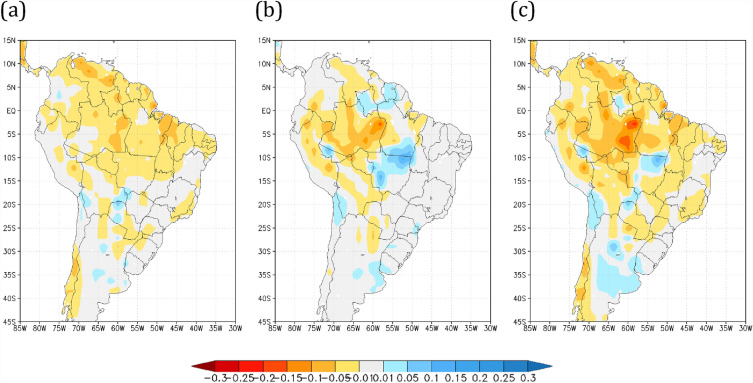
Annual mean soil moisture of the surface layer (in dim. 0–1) anomalies relative to the HiFo. Annual mean soil moisture (in dim. 0–1) anomalies relative to the HiFo experiment for the (**a**) R8Fo, (**b**) HiSa, and (**c**) R8Sa scenarios. Maps made by COLA GrADS v2.0.

Several studies^59–61^ have suggested that climate change can affect both groundwater content and recharge, which can threaten water availability for rural communities and cities. The combined effects of precipitation and temperature changes for global warming and Amazon savannization experiments, discussed above, reduced surface soil moisture throughout South America (Fig. 6). The effects of global warming (R8Fo) alone decreased soil moisture by up to 10% relative to the forested historical (HiFo) run, most prominently over northern South America and the Amazon Basin (Fig. 6a). The HiSa experiment reduces soil moisture by up to 15% relative to the HiFo case over the eastern portion of the Amazon Basin (Fig. 6b). However, when considering the combined effects of global warming and Amazon savannization, soil dryness becomes even more prominent, spreading to almost the entire Brazil and neighboring countries (Fig. 6c). Other studies^21,62^ have shown that global warming and reduced precipitation are among the main drivers for the reduction of total soil moisture content, aggravating the disruption of the global water cycle and enhancing the variability of extreme meteorological disasters.

**Figure 7:**
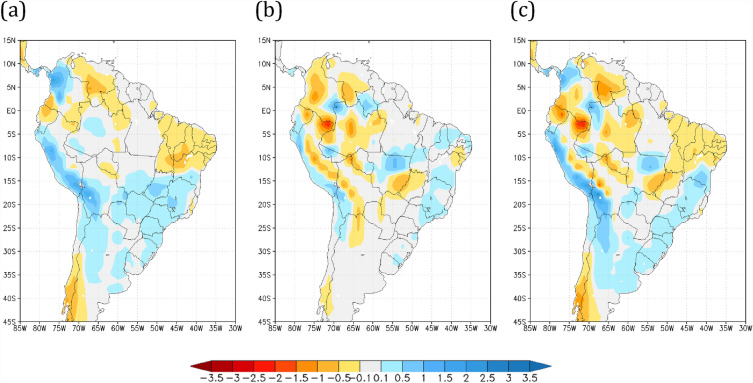
Anomalies in the annual mean surface runoff (in mm/day) relative to the HiFo control. Annual mean surface runoff (in mm/day) anomalies relative to the HiFo experiment for the (**a**) R8Fo, (**b**) HiSa, and (**c**) R8Sa scenarios. Maps made by COLA GrADS v2.0.

We next examined the effects of global warming and savannization on superficial runoff. When global warming alone is considered, runoff decreases over northern Brazil and increases over Southern Brazil (Fig. 7a), consistent with results from other studies^63–65^; however, consistent with the reduced southward moisture advection due to the Amazon savannization discussed above, the surface runoff reduction extends from the Amazon region to Central-West Brazil (Fig. 7b). The composite effects of global warming and Amazon savannization on surface runoff are shown in Fig. 7c. The reduced runoff in the Central-West region associated with Amazon savannization observed in both the R8Fo and R8Sa experiments might have important downstream consequences, e.g., adverse effects on hydroelectric power generation. The spatial distribution of soil moisture and runoff for the HiFo run are shown in Supplementary Figures S7 and S8.

## Conclusions

In this study, we examined the separate and combined effects of vegetation cover changes over the Amazon Basin and global warming on rainfall, dry period length, air temperature, and humidity over South America, with a focus on Brazil. We examined the worst-case scenario, in which the entire Brazilian Amazon rainforest is replaced with savannah vegetation. The results of our Amazon Basin savannization simulations reveal a severe reduction in precipitation over the Amazon and Central-West regions, and smaller reduction over the southeastern region of Brazil. Our study demonstrated that reduced rainfall over the regions to the south of the Amazon is in part due to a lack of southward moisture transport from the Amazon region when rainforest is replaced with savannah vegetation. The combined effects of Amazon savannization and global warming resulted in the most dramatic increase of dry season length and temperature over the Amazon region, and the reductions of rainfall, soil moisture, and surface runoff, extending from the Amazon to the southern portions of Brazil. The decreased rainfall and soil moisture shown over the Central-West region are in part a consequence of reduced southward moisture transport from the Amazon and increased water loss due to higher air temperatures.

## Methods

### Meteorological data and precipitation bias correction

The precipitation data is from the Global Precipitation Climatology Project (GPCP) as described in^66^. The GPCP precipitation dataset provides daily rainfall accumulation globally on a one-degree grid in latitude and longitude. The global daily data set was linearly interpolated for spatial resolution of the BESM model (1.85°). The historical data period from 2001 to 2020 was used to guide the current conditions and bias correction procedures. Simulated daily precipitation was corrected using the quantile-based mapping method^67–69^, based on the statistical moments of cumulative distribution functions (CDF) of observations and model simulations, mapping the projected climate variables to the observed CDFs with Eq. (1):

1 $$x_{m - p} = F^{{ - {1}}}{}_{o - c} \left[ {F_{m - c} \left( {x_{m - c} } \right)} \right],$$

where *F*^*−*1^_*o*−*c*_ is the quantile function corresponding to observations *o* for a historic training period *c*, *F*_*m*−*c*_ is the CDF of model simulated fields *m* for a historic training period, and *p* is the future projection climate fields. This method assumes that the relationship between the model-simulated and observed values during the training (forested historic) period also apply to the future period. A common approach is to solve Eq. (1) using the empirical CDF of observed and modeled values. The empirical cumulative distribution functions of observations and historic forested simulation were constructed for each grid point by daily precipitation values for each month of the year, during the 20 years (2001–2020) for the GPCP data and the 28 years (1983–2010) for historical simulation. The CDF was formed by discrete histograms with 0.1 mm/day intervals from 0 to 600 mm/day. The method was applied to the set of experiments to obtain a new corrected daily time series over 28 years.

### Daily maximum air temperature correction

The observational data used in this study were from the reanalysis of the European Centre for Medium-Range Weather Forecasts (ECMWF) ERA5^70^. Hourly data fields of 2 m temperature were linearly interpolated to match the spatial resolution of the BESM model. Historical period data from 1981 to 2010 were used as the current conditions for the bias correction of the model outputs. Observational daily maximum air temperature values from the reanalysis were used for comparisons with the simulation of the forested Historical period and bias correction procedures. The method used to correct the temperature bias was based on variable normalization. The Standard Normal Distribution-based scaling used in the experiment is a simple approach that matches only the first and second moments of the observations and model distributions^71^. The correction was applied separately for each month to account for possible seasonal cycle changes in the climatological differences. For a given calendar month *m* and a given grid cell *i*, the scaling parameters are the daily maximum air temperature, evaluated by the 28-year mean (*M*_*mi,m*_ and *M*_*oi,m*_ for model and observations, respectively) and the standard deviation (*S*_*mi,m*_ and *S*_*oi,m*_ for model and observations, respectively). For each model temperature *T*_*i*_ from a particular month (subscript omitted), the scaled model $T_{i}^ {\prime}$ is then given by $T_{i}^ {\prime} \, = \, \left( {T_{i} - M_{mi,m} } \right)S_{oi,m} /S_{mi,m} + M_{oi,m}$. The means and standard deviations of the forested Historical experiment and the reanalysis were used to correct the computed daily maximum air temperature bias for the savannization and RCP experiments. The use of the forested Historic parameters to correct for bias in the savannization and RCP experiments is valid given the experimental boundary conditions, although the physics and dynamics of the model were not altered.

### Coupled ocean–atmosphere land ice model

The savannization and warming scenarios simulations were produced by the Brazilian Earth System Model, version 2.5 coupled ocean–atmosphere model (BESM-OA2.5) developed at the Brazilian National Institute for Space Research (INPE). BESM-OA2.5 comprises the Brazilian Global Atmospheric Model (BAM) of the Center for Weather Forecasting and Climate Studies (CPTEC/INPE) and the Geophysical Fluid Dynamics Laboratory (GFDL) Modular Ocean Model version 4p1 (MOM4p1) of the National Oceanic and Atmospheric Administration (NOAA). More details are described elsewhere^47,48^.

The atmospheric component BAM, described by^72^, adopted the horizontal grid resolution truncated at triangular wavenumber 62 (approximately 1.875 × 1.875 degrees of resolution at the equator) and 28 sigma vertical levels. The surface model is SSib^73^ for the heat flux and soil condition calculations. The SSib is a static vegetation surface model in which the surface and soil characteristics are seasonally parameterized and spatially distributed using a map with 13 types of ground surface cover. Regional changes in this map allow for simulating changes in surface characteristics, such as Amazon Basin savannization. This is the basis of the experimental design described in the next section. One improvement incorporated in this version is the representation of the wind, humidity, and temperature in the surface layer using a formula described by Jiménez et al.^74^, with more details in^48^. Representation of the near-surface conditions is important in the simulation of Amazonian climate change.

### Experimental design

The numerical experiments were performed with the BESM-OA2.5 using two surface cover boundary conditions: (1) the original SSib vegetation map^75^, where the Amazon Basin cover corresponds to “Broad-leaf evergreen trees” (hereinafter referred to as Forest); and (2) a change in the Brazilian Amazon Basin vegetation cover to “Broad-leaf trees with ground cover” (hereinafter referred to as Savannah). In the context of our numerical experiments, savannization means the change of the surface parameters; albedo, roughness length and stomatal resistance from tropical forests to savannah parameters over the Brazilian Amazon region. Maps of the SSib cover types are shown in Supplementary Fig. 1S. As part of the experimental design, only the Brazilian Amazon rainforest was considered.

The model simulations follow the CMIP5 experimental design protocol^76^. Three sets of experiments were performed: one Historical over the period 1981–2010 (30 years), forced by the observed historical atmospheric equivalent CO_2_ concentration (greenhouse gas only), and two warming scenarios over the period 2071–2100 (30 years), forced by the time-dependent changes in GHG levels projected by the Representative Concentration Pathway 8.5 (RCP8.5). These two period sets were run with two surface conditions, yielding a total of 4 experiments.

### Calculation of dry season distribution

The dry season length was examined as the distribution of consecutive dry days. At each grid point, the 10,220-day (28-year) time series was followed by identifying the length of consecutive dry day periods. Similar to studies that calculate the length of the rainy season^17,77^, the beginning and end of dry periods are determined by the precipitation threshold. To identify dry periods that could affect crops, different thresholds were used for the beginning and end of dry periods. Specifically^78^, defined a precipitation threshold of 2.5 mm/day, which corresponds to the amount necessary for soybean seedlings to survive and grow; therefore, daily precipitation rates below the threshold of 2.5 mm/day indicate the beginning of a dry period. Arvor et al.^79^ defined a precipitation threshold of 5.1 mm/day, which represents the water demand of soybeans at the beginning of the vegetative cycle. Exceeding this threshold identifies the end of the dry period. The precipitation threshold of 5.1 mm/day is also consistent with late-season maize evapotranspiration^80^. The length of the dry season is calculated as the frequency distribution of the number of consecutive dry days, in ten days' classes for each grid point.

### Supplementary Information

Supplementary Information.

## Data Availability

The datasets used and/or analyzed during the current study are available from the corresponding author upon reasonable request.
