# Supplementary material for: Amazon savannization and climate change are projected to increase dry season length and temperature extremes over Brazil
Source: Sci Rep. 2024 Mar 1;14:5131. doi: 10.1038/s41598-024-55176-5 (PMC11319773; doi:10.1038/s41598-024-55176-5)
Supplement: Supplementary file 1 — Supplementary Information. [file 41598_2024_55176_MOESM1_ESM.docx]

**Amazon savannization and climate change are projected to increase dry season length and temperature extremes over Brazil**

*Marcus Jorge Bottino¹, Paulo Nobre¹, Emanuel Giarolla¹, Manoel Baptista da Silva Jr.¹, Vinicius Buscioli Capistrano², Marta Malagutti¹, Jonas Tamaoki¹, Beatriz Fátima Alves de Oliveira³, Carlos Afonso Nobre^4^*

| (a)  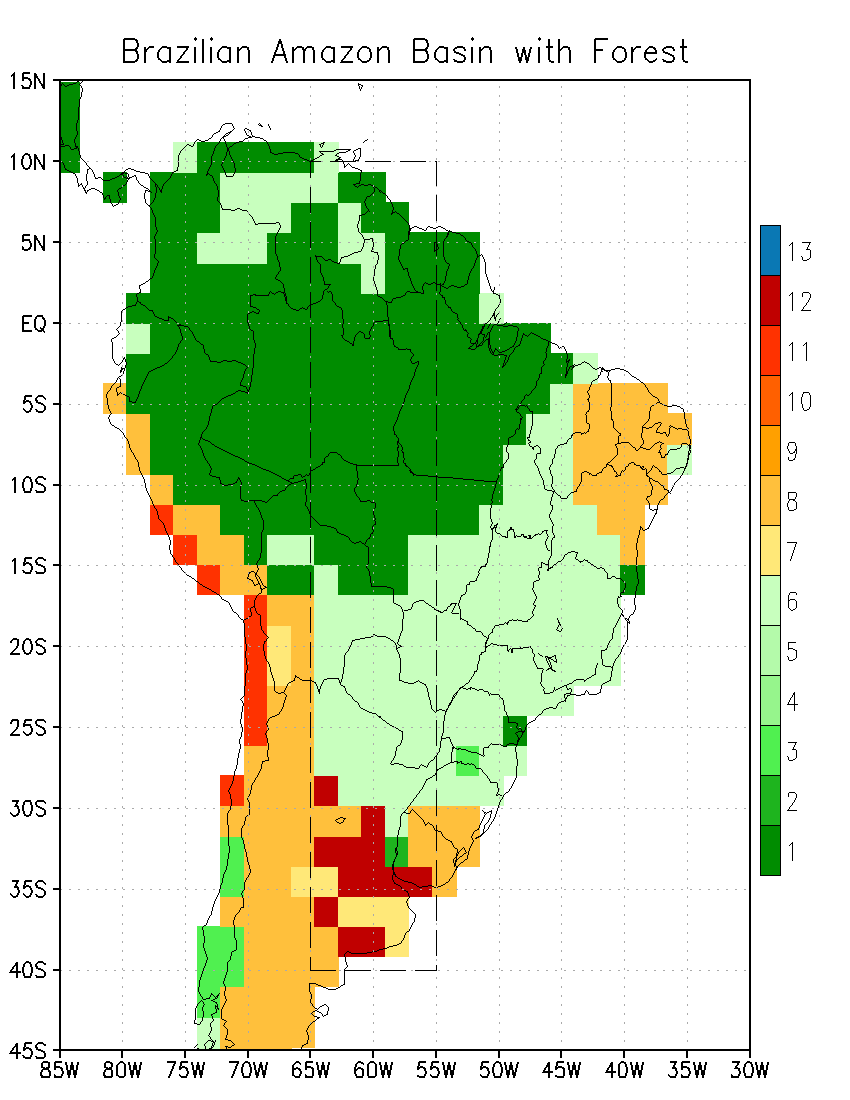 | (b)  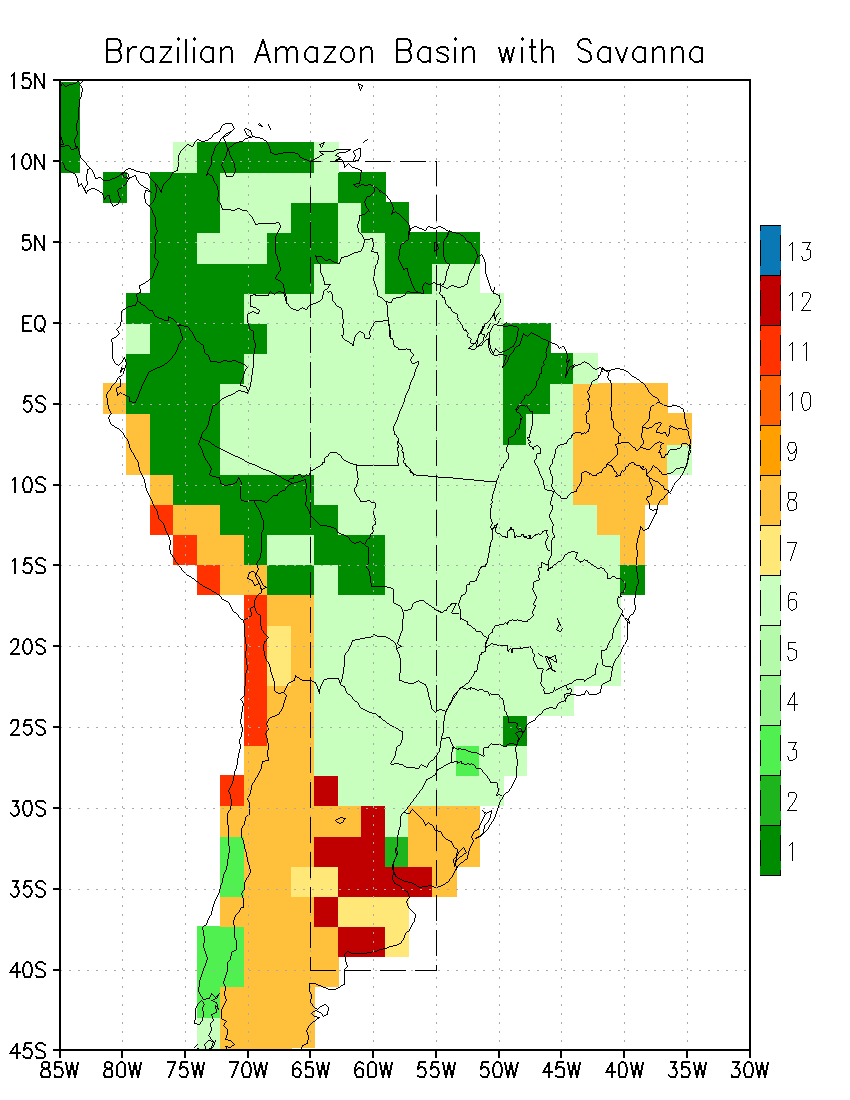 |
| --- | --- |
| (c)  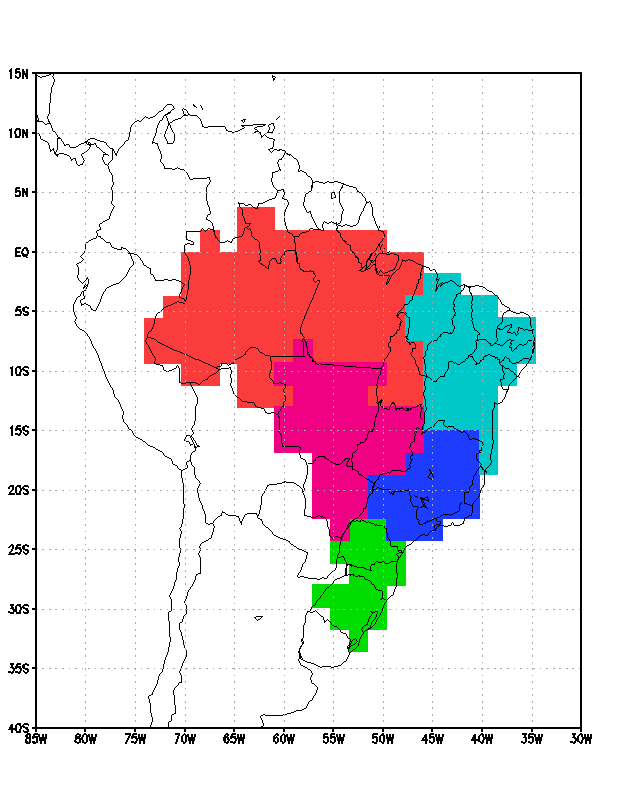 |  |

**Figure S1.** **Maps of SSib cover types of experiments and regions of Brazil.** Maps of SSib cover types of scenarios experiments for (a) Forest (type 1), (b) Savanna (type 6), and (c) regions of Brazil masks. The dashed rectangle represents the region used to calculate the meridional humidity advection. Maps made by COLA GrADS v2.0.

| 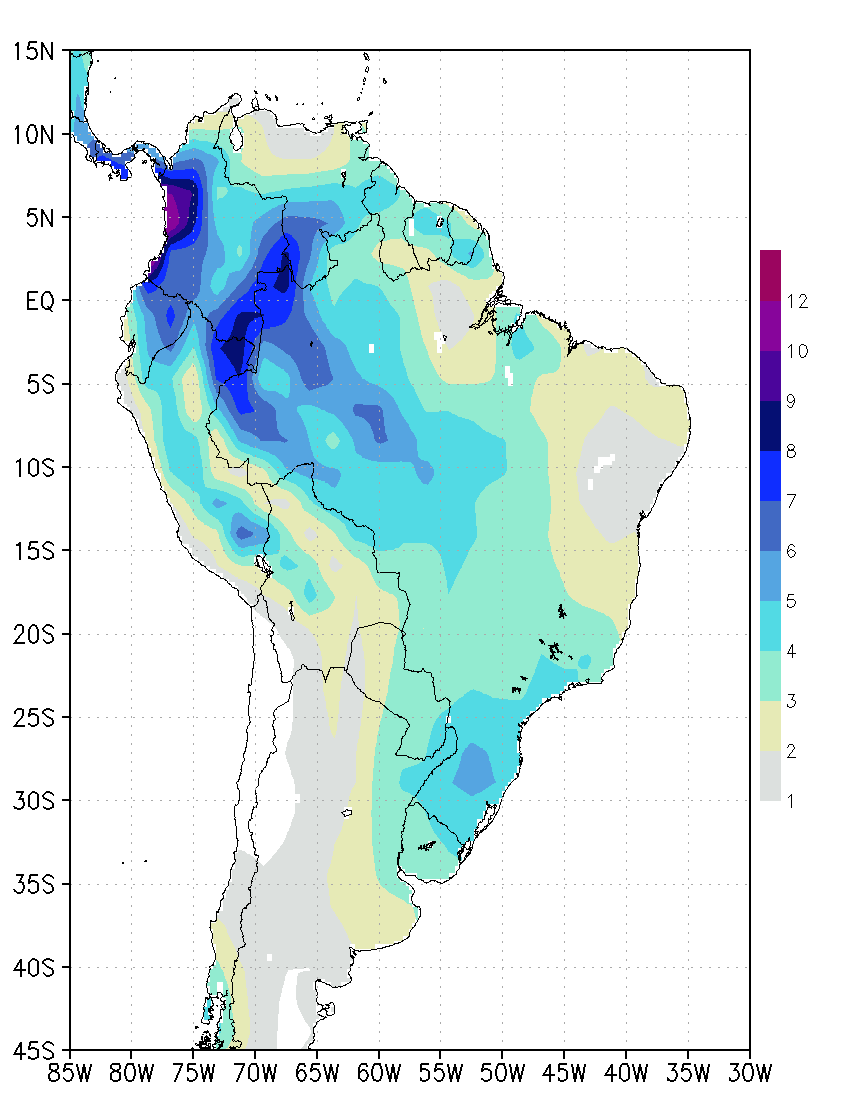 |
| --- |

**Figure S2.** **Annual mean precipitation.** Annual mean precipitation (in mm/day) for historical forested (HiFo, 1983–2010) control run. Map made by COLA GrADS v2.0.

| 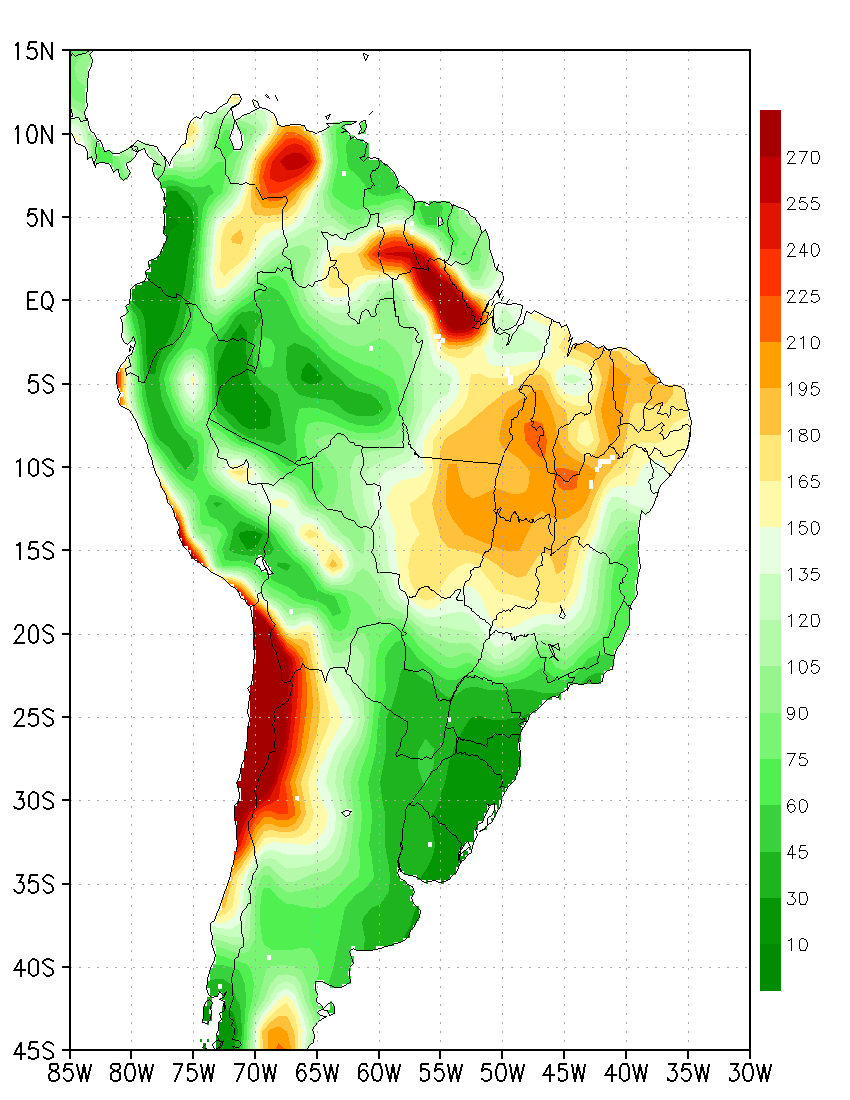 |
| --- |

**Figure S3.** **Dry season length (in days).** The dry season length of the tail distribution of the largest 10% of the number of consecutive dry days (in days) for historical forested (HiFo) control run. Map made by COLA GrADS v2.0.

| (a)  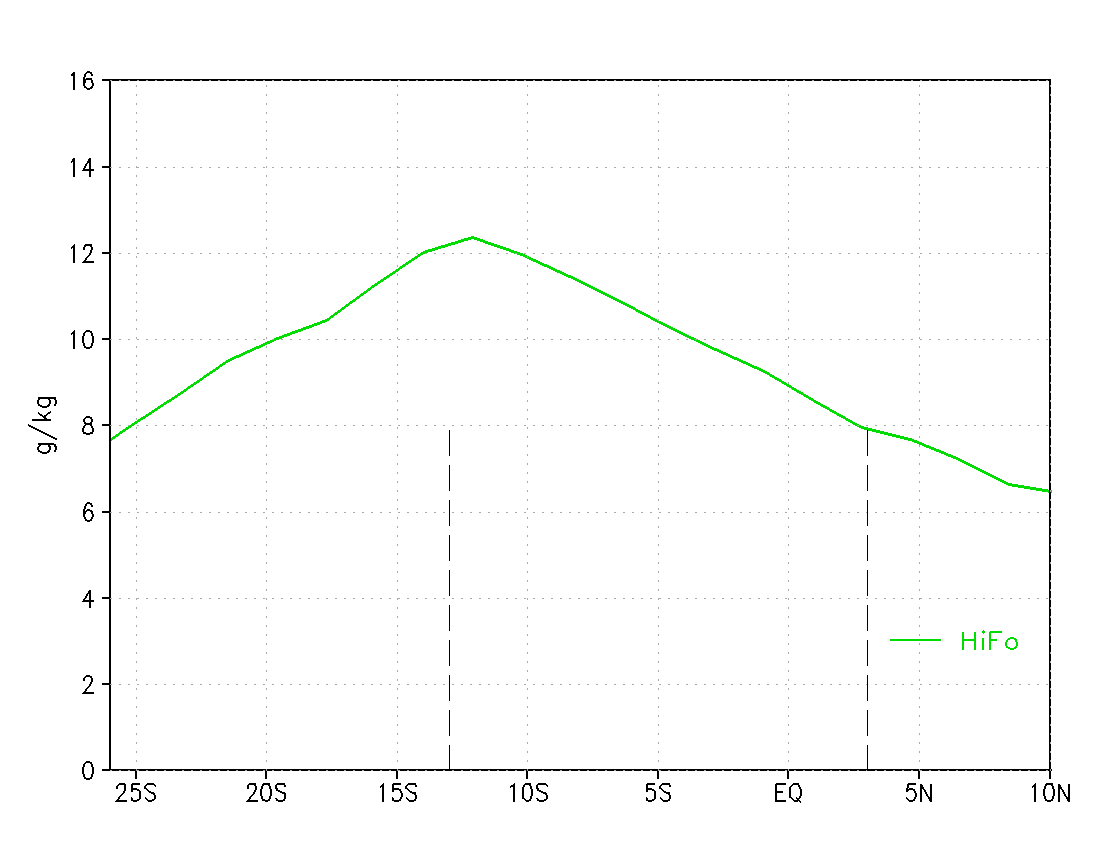 | (b)  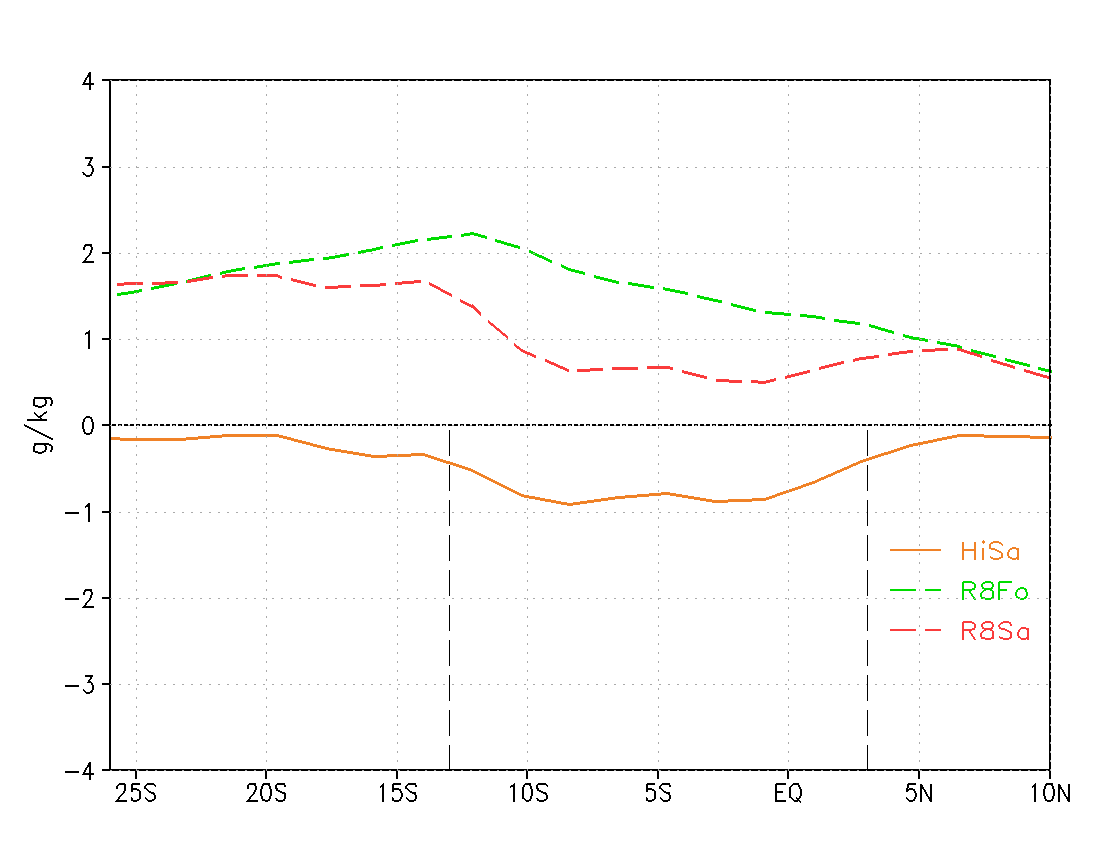 |
| --- | --- |
| (c)  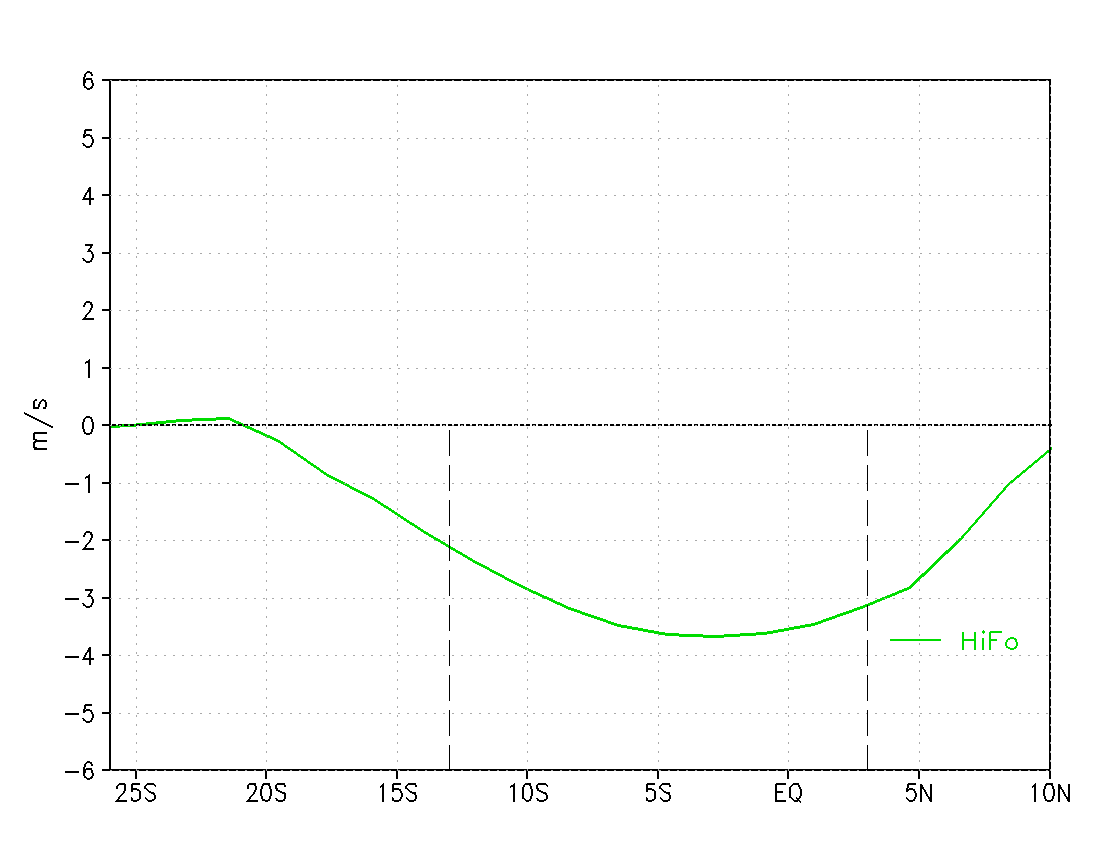 | (d)  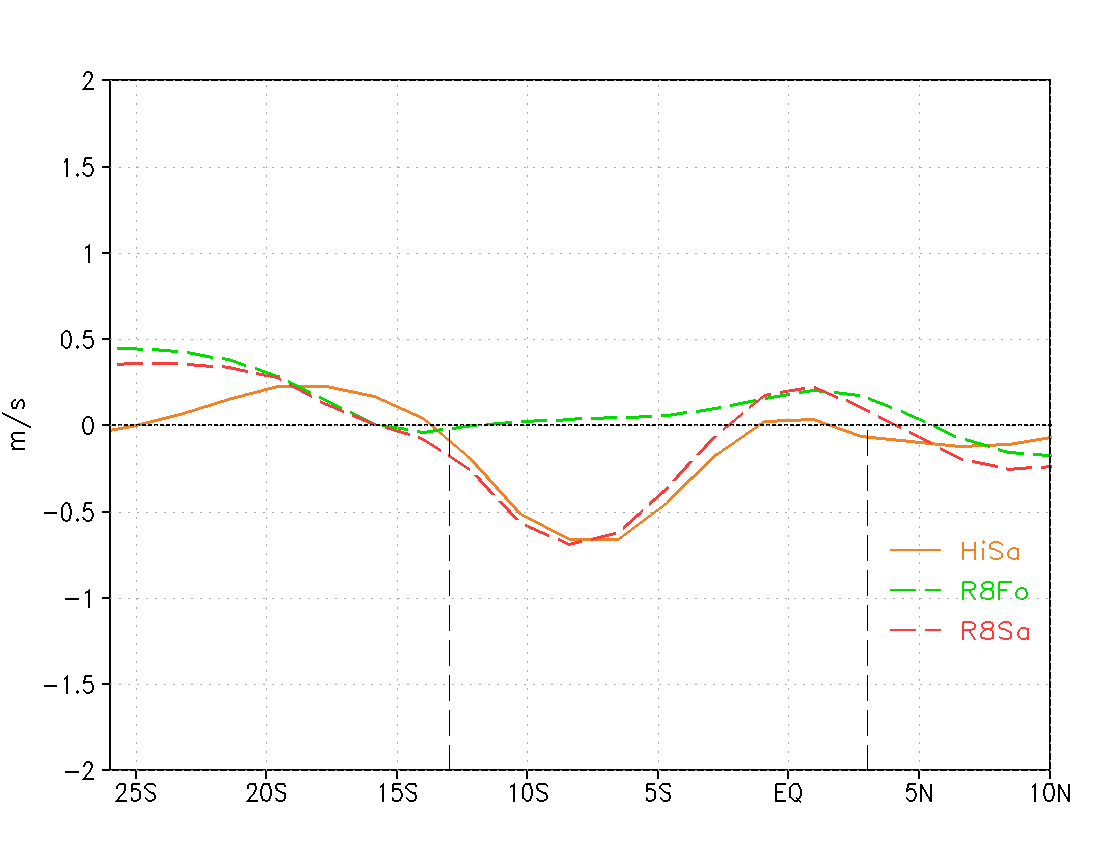 |
| (e)  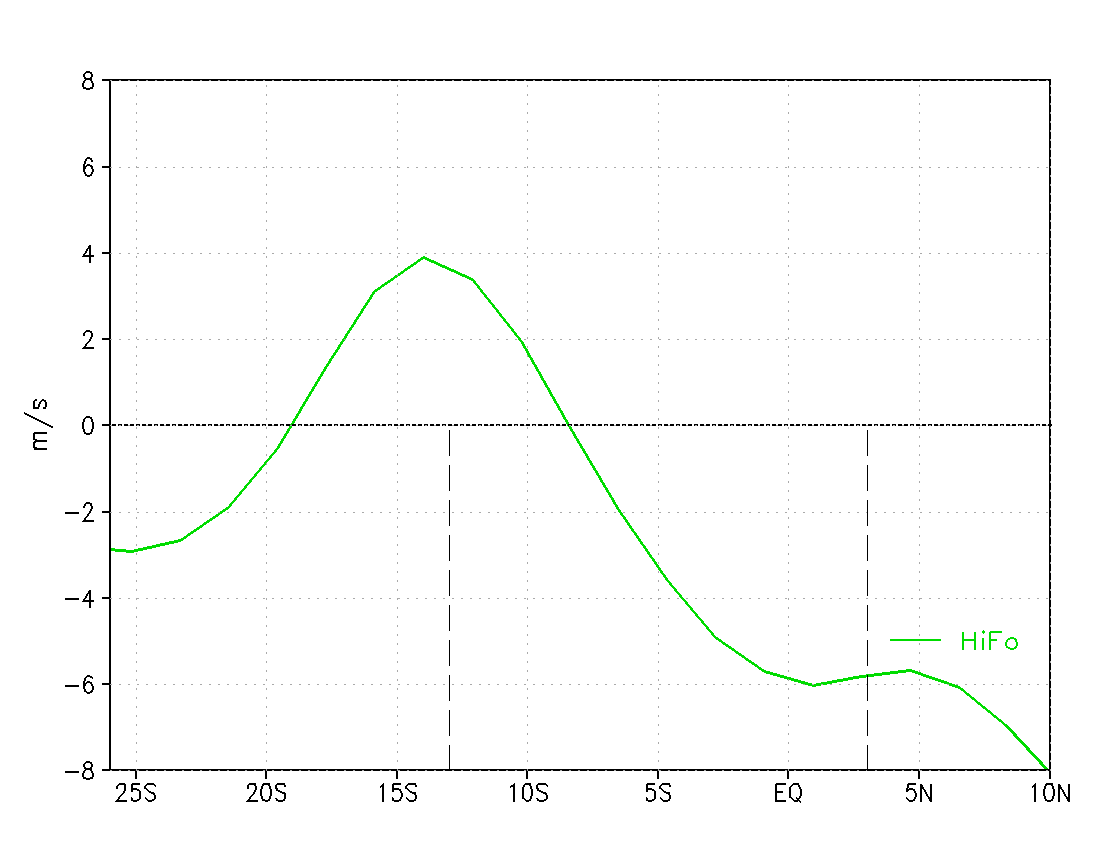 | (f)  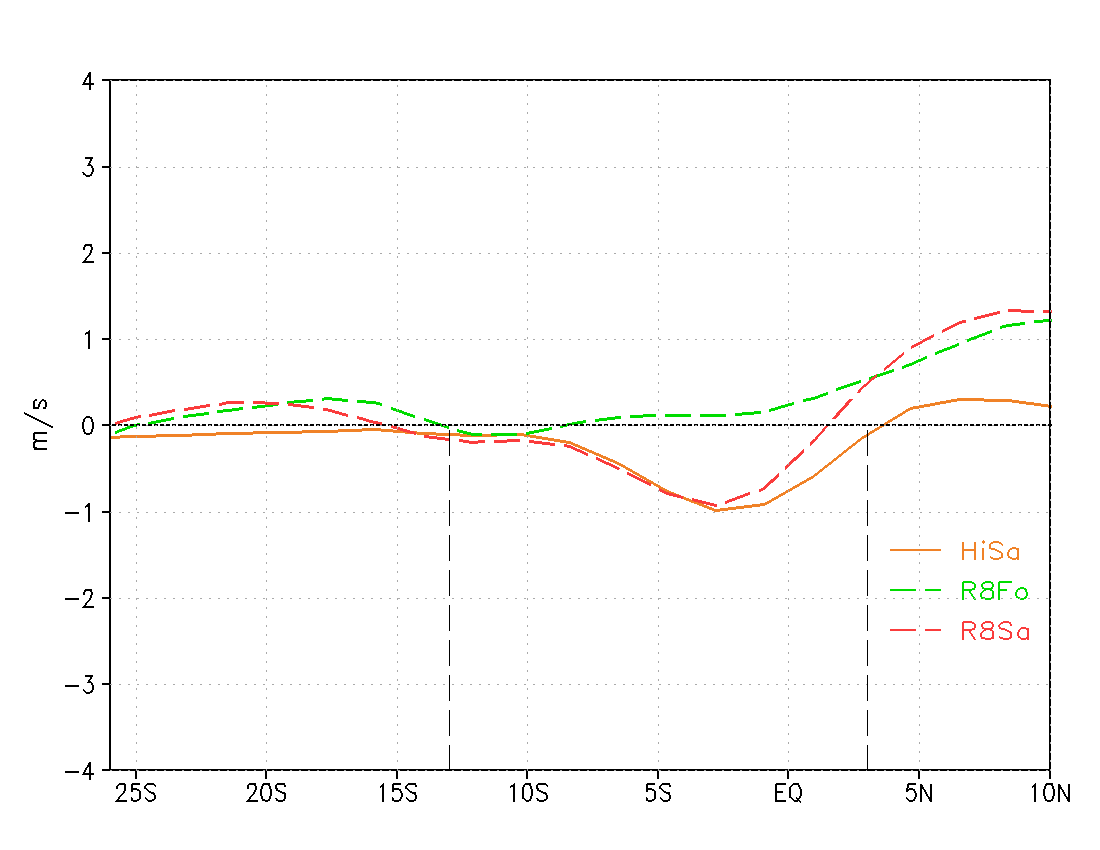 |

**Figure S4.** **Low troposphere meridional conditions.** Low troposphere vertically averaged (975-700 hPa) specific humidity (a, b, in g/kg), meridional wind (c, d, in m/s) and zonal wind (e, f, in m/s), mean DJFM months, for (a, c, e) HiFo and (b, d, f) the scenarios differences respect to HiFo. The Amazon Basin latitude range is delimited by vertical black dashed lines.

| 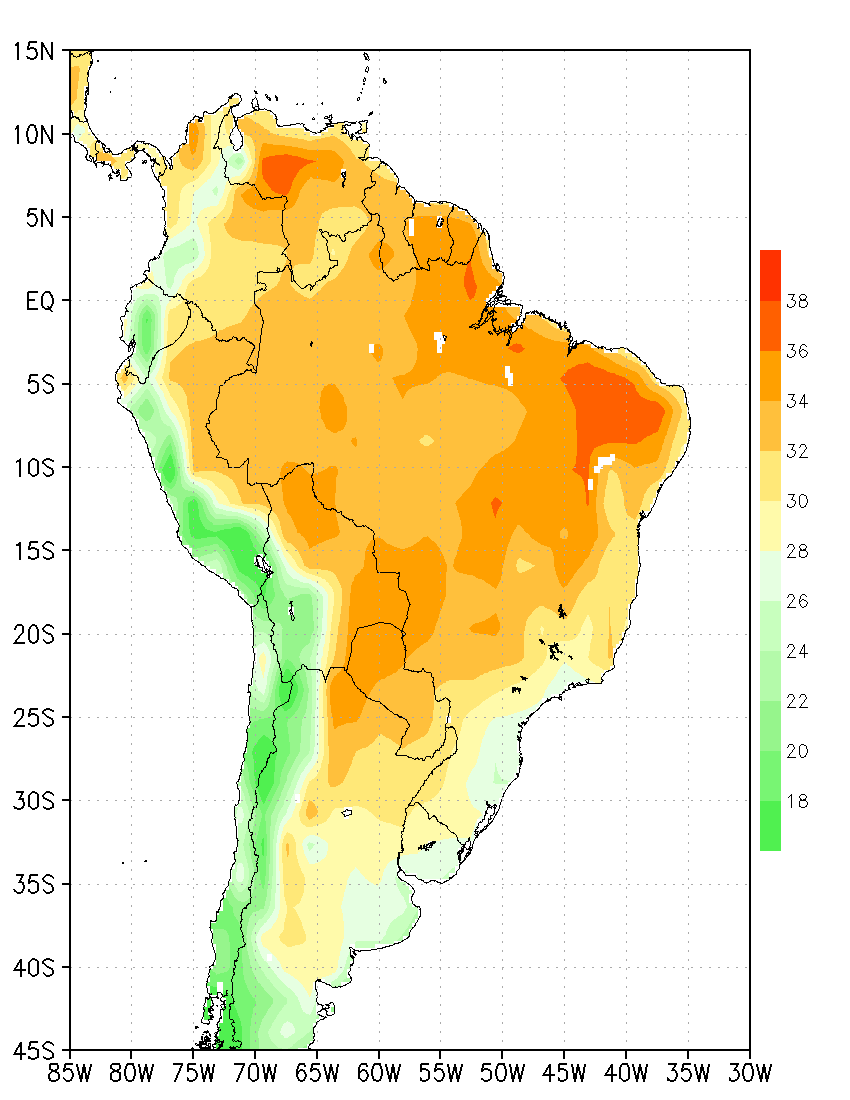 |
| --- |

**Figure S5.** **Climatological daily maxima air temperature (in °C).** Climatological daily maxima air temperature (in °C) for the warmest month for historical forested (HiFo) control run. Map made by COLA GrADS v2.0.

| (a)  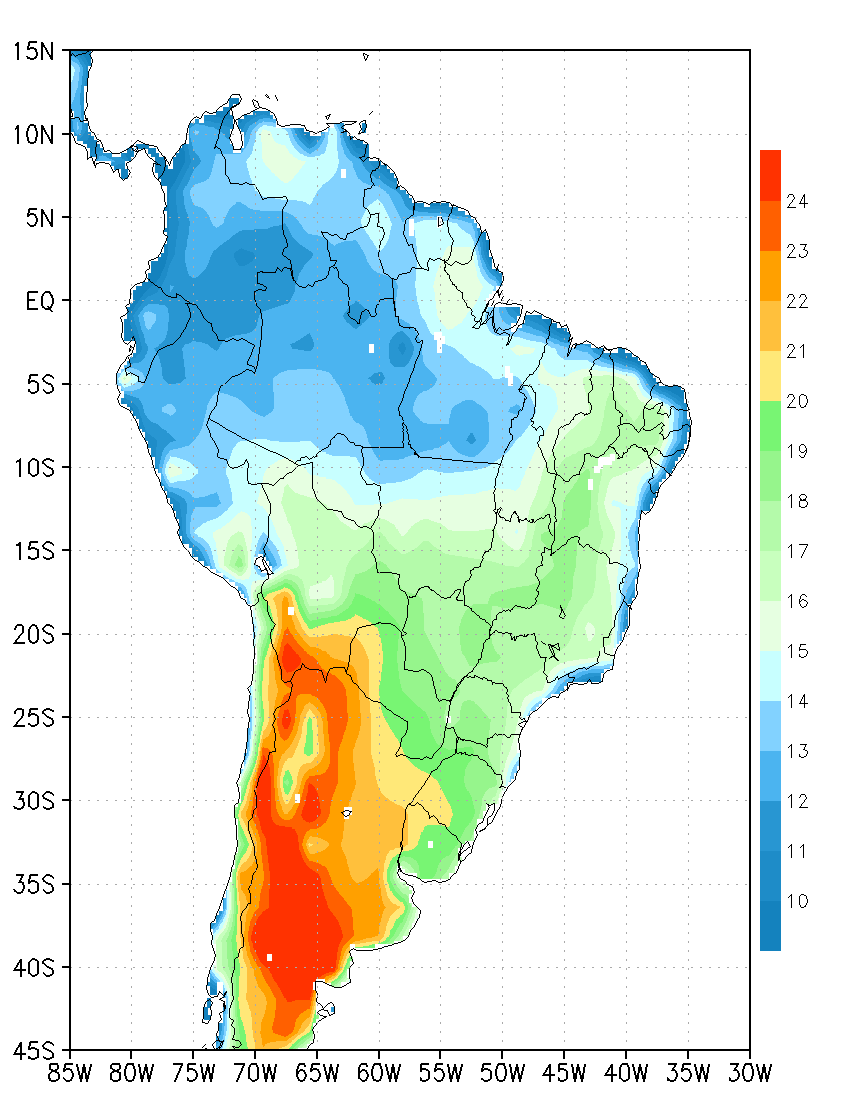 | (b)  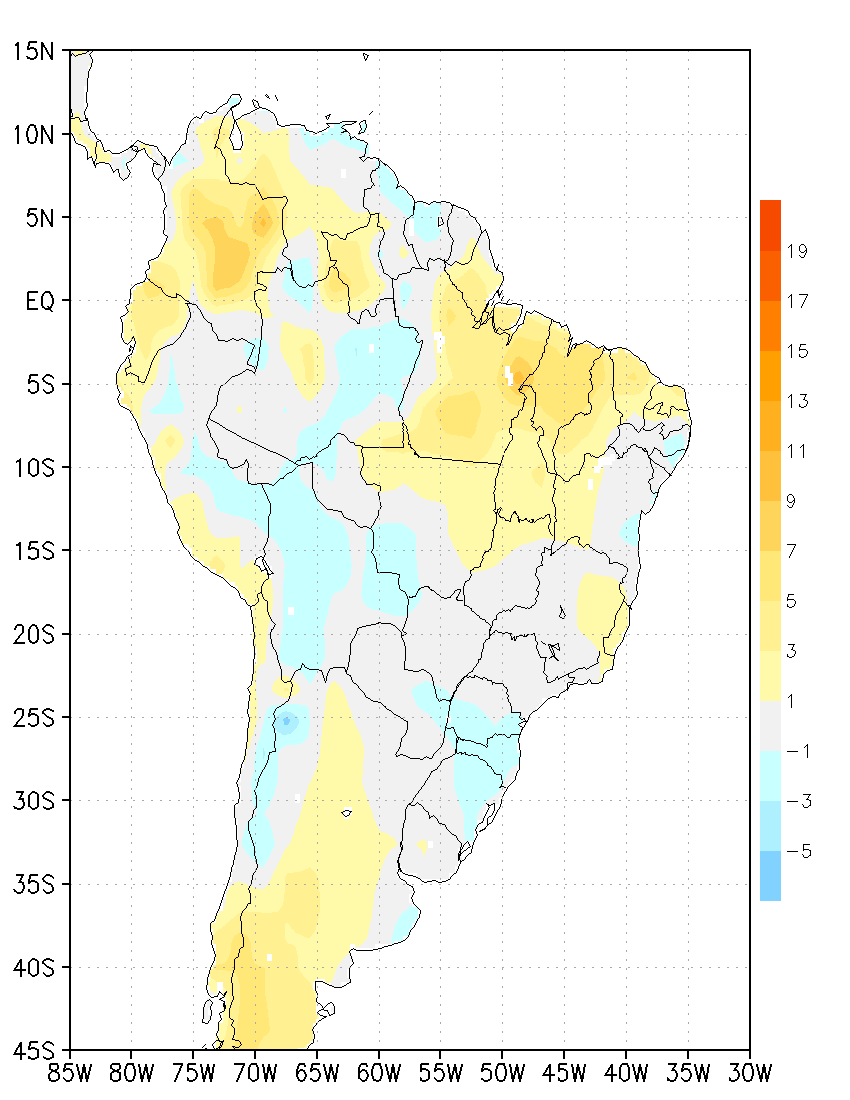 |
| --- | --- |
| (c)  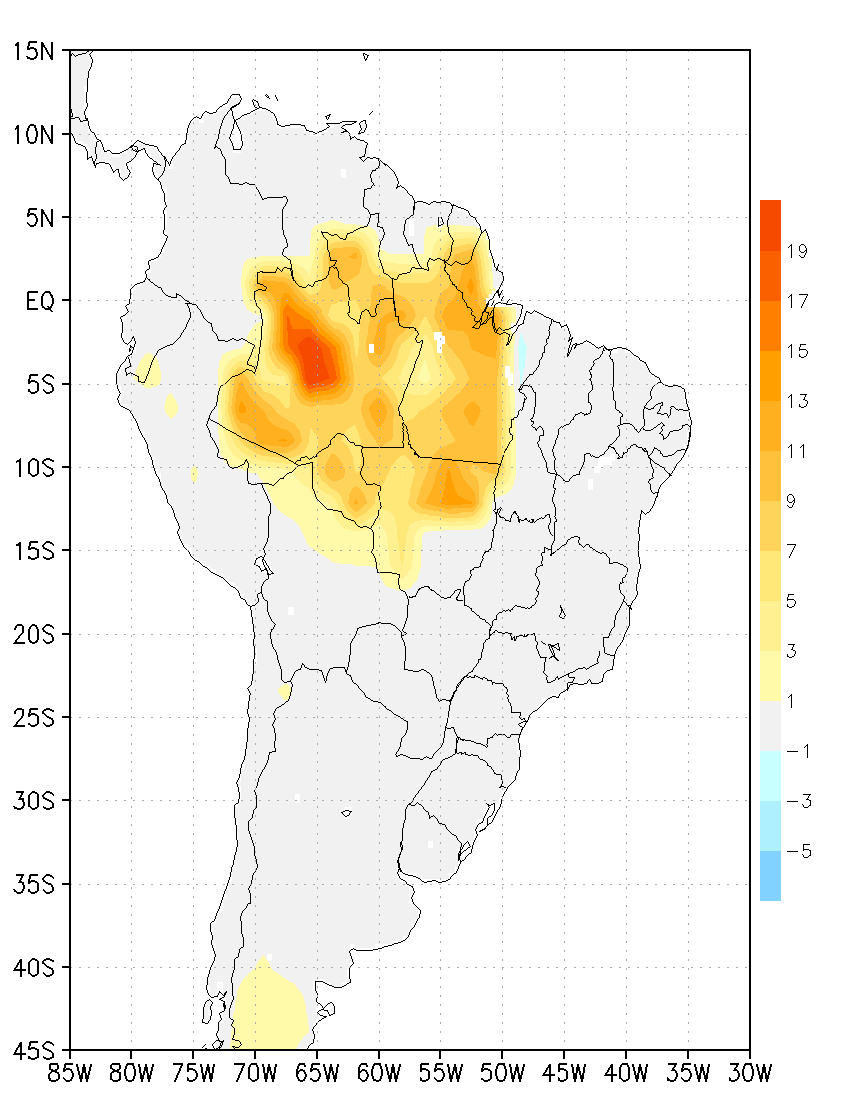 | (d)  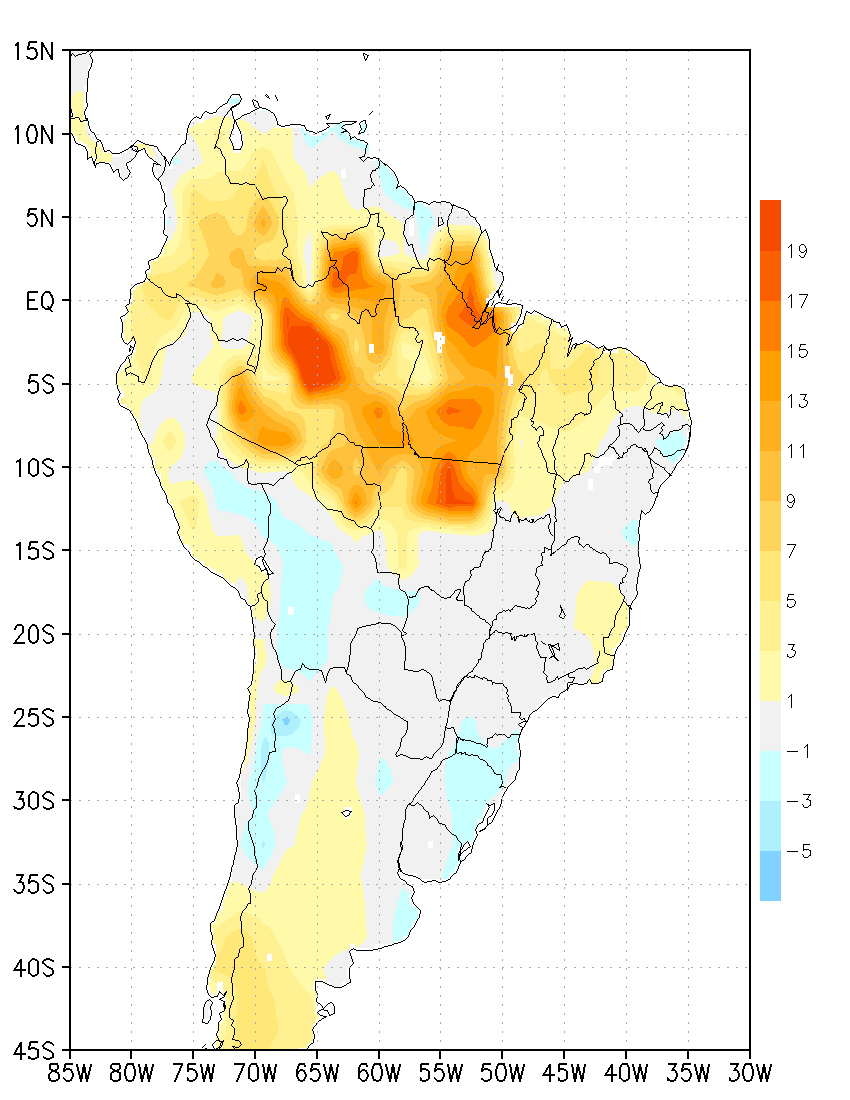 |

**Figure S6.** **Climatological seasonal air temperature difference (in °C).** Climatological air temperature difference from the daily maximum of the warmest month and the daily minimum of the coldest month (in °C), for (a) HiFo and the differences (b) R8Fo, (c) HiSa and (d) R8Sa experiments relative to HiFo. Maps made by COLA GrADS v2.0.

| **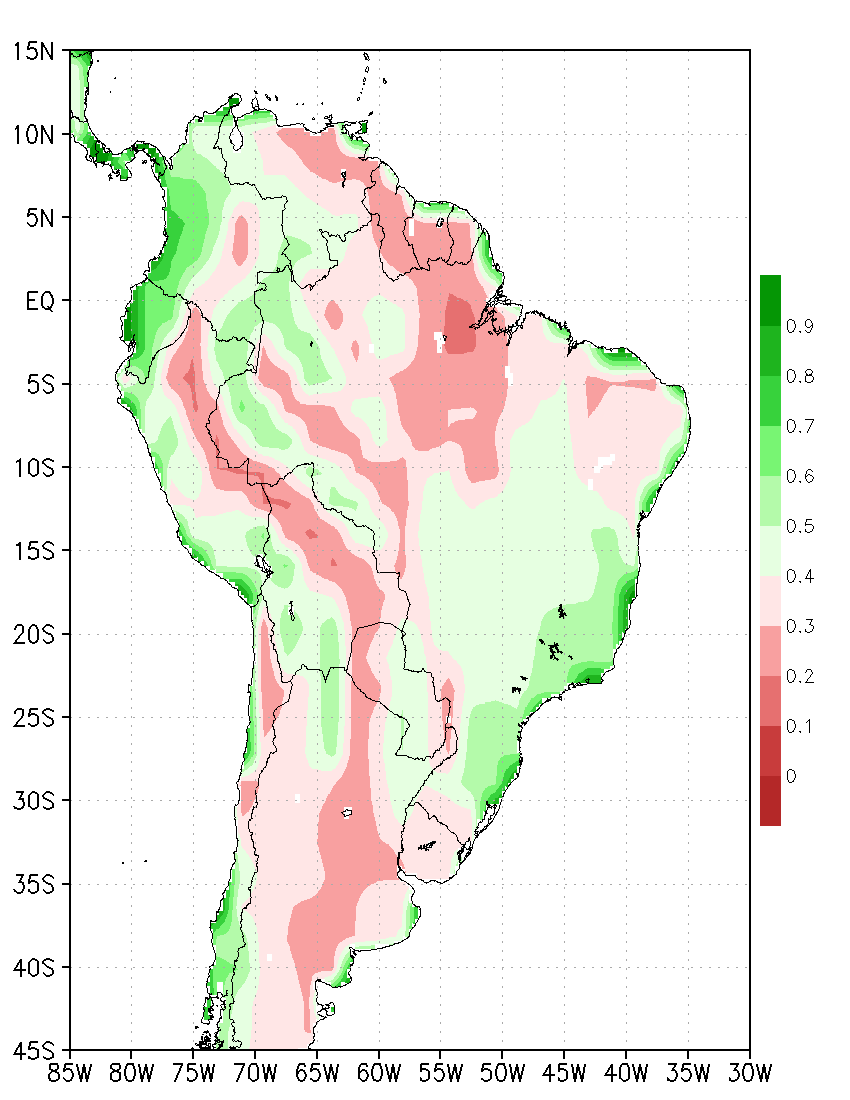** |
| --- |

**Figure S7.** **Annual mean soil wetness of surface layer (in dim. 0-1).** Annual mean soil wetness (in dim. 0-1) of surface layer for historical forested (HiFo) control run. Map made by COLA GrADS v2.0.

| **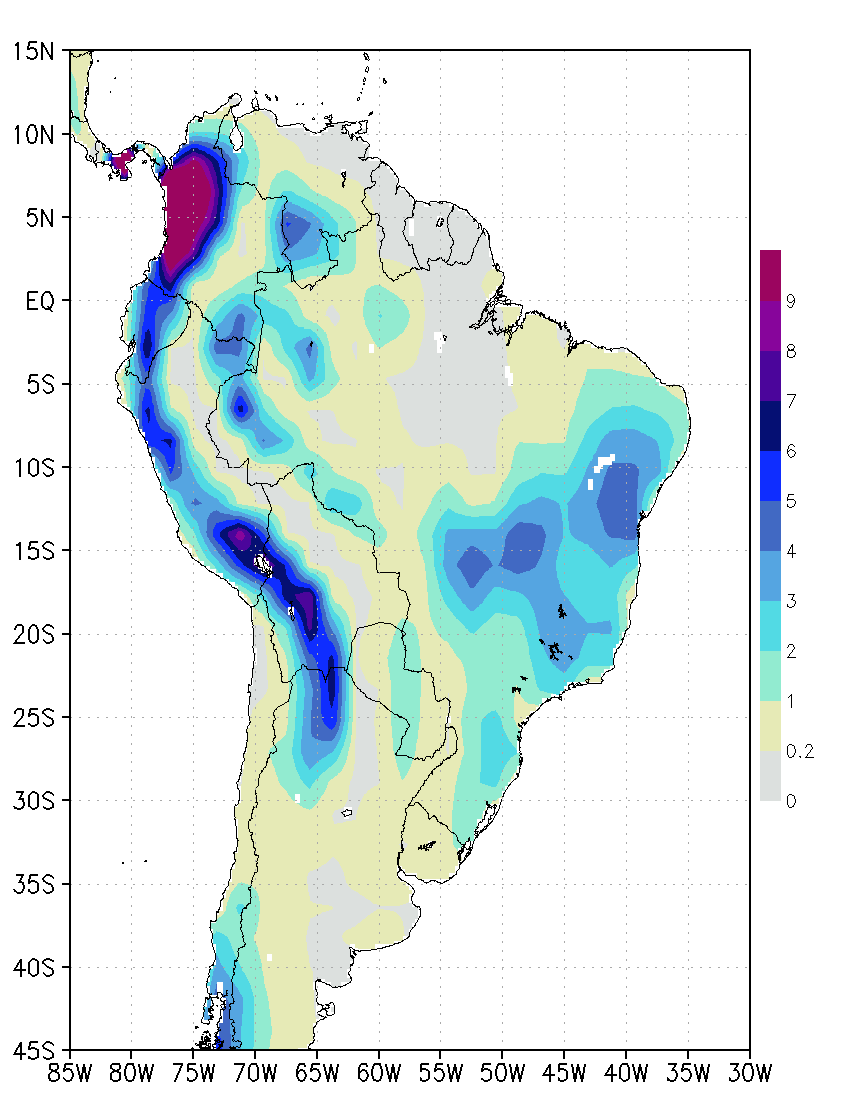** |
| --- |

**Figure S8.** **Annual mean surface runoff (in mm/day).** Annual mean surface runoff (in mm/day) for historical forested (HiFo) control run. Map made by COLA GrADS v2.0.
